# Developing a MEMS Device with Built-in Microfluidics for Biophysical Single Cell Characterization

**DOI:** 10.3390/mi9060275

**Published:** 2018-06-01

**Authors:** Yuki Takayama, Grégoire Perret, Momoko Kumemura, Manabu Ataka, Samuel Meignan, Stanislav L. Karsten, Hiroyuki Fujita, Dominique Collard, Chann Lagadec, Mehmet Cagatay Tarhan

**Affiliations:** 1 Laboratory for Integrated Micro Mechatronic Systems (LIMMS/CNRS-IIS), Institute of Industrial Science, The University of Tokyo, 4-6-1 Komaba, Meguro-ku, Tokyo 153-8505, Japan; yktkym@iis.u-tokyo.ac.jp (Y.T.); gperret@iis.u-tokyo.ac.jp (G.P.); 2 Univ. Lille, CNRS, Centrale Lille, ISEN, Univ. Valenciennes, UMR 8520-IEMN, 59652 Villeneuve d’Ascq, France; 3 CNRS/IIS/COL/Lille University SMMiL-E Project, CNRS Délégation Nord-Pas de Calais et Picardie, 2 rue de Canonniers, Lille, Cedex 59046, France; ataka@iis.u-tokyo.ac.jp (M.A.); hfujita@iis.u-tokyo.ac.jp (H.F.); chann.lagadec@inserm.fr (C.L.); 4 Graduate School of Life Science and Systems Engineering, Kyushu Institute of Technology, 2-4 Hibikino, Wakamatsu-ku, Kitakyushu-shi, Fukuoka 808-0196, Japan; momo@life.kyutech.ac.jp; 5 Centre for Interdisciplinary Research on Micro-Nano Methods, Institute of Industrial Science, The University of Tokyo, 4-6-1 Komaba, Meguro-ku, Tokyo 153-8505, Japan; 6 Tumorigenesis and Resistance to Treatment Unit, Centre Oscar Lambret, Université de Lille, 3 rue Frédéric Combemale, 59000 Lille, France; s-meignan@o-lambret.fr; 7 INSERM U908 Laboratory, Lille University—Science and Technologies, Building SN3, 59655 Villeneuve d’Ascq, France; 8 NeuroInDx, Inc., 20725 S Western Ave #100, Torrance, CA 90501, USA; skarsten@neuroindx.com

**Keywords:** single cell analysis, biophysical cell characterization, bioMEMS, microfluidics, MEMS design

## Abstract

This study combines the high-throughput capabilities of microfluidics with the sensitive measurements of microelectromechanical systems (MEMS) technology to perform biophysical characterization of circulating cells for diagnostic purposes. The proposed device includes a built-in microchannel that is probed by two opposing tips performing compression and sensing separately. Mechanical displacement of the compressing tip (up to a maximum of 14 µm) and the sensing tip (with a quality factor of 8.9) are provided by two separate comb-drive actuators, and sensing is performed with a capacitive displacement sensor. The device is designed and developed for simultaneous electrical and mechanical measurements. As the device is capable of exchanging the liquid inside the channel, different solutions were tested consecutively. The performance of the device was evaluated by introducing varying concentrations of glucose (from 0.55 mM (0.1%) to 55.5 mM (10%)) and NaCl (from 0.1 mM to 10 mM) solutions in the microchannel and by monitoring changes in the mechanical and electrical properties. Moreover, we demonstrated biological sample handling by capturing single cancer cells. These results show three important capabilities of the proposed device: mechanical measurements, electrical measurements, and biological sample handling. Combined in one device, these features allow for high-throughput multi-parameter characterization of single cells.

## 1. Introduction

Cells in a sample solution can be largely heterogeneous, consisting of various different sub-populations, even if they originate from the same tumor or cell line. Immunochemistry has been an effective means to investigate sub-populations within a cell solution especially based on specific membrane protein expressions, designated as “cluster of differentiation” (CD). Highly specific immunoaffinity-based assays represent a powerful analysis method, especially with the development of high-throughput cell cytometry. However, the use of biomarkers is still an important limitation due to practical reasons, such as availability of surface markers and corresponding antibodies and heterogeneity between specimens regarding combined marker expressions. Therefore, routine clinical tests for early diagnosis would benefit from cost and time-efficient alternatives.

Cell shape and structural integrity significantly influence many biological processes. Therefore, the physical properties of cells may potentially be used to reflect the state of their health [1]. This connection between biophysics and disease has been attracting scientific research attention, especially for oncological studies [2,3,4,5,6,7] where diseased cells proliferate “uncontrollably” and disrupt the organization of tissue. When the physical properties of a cell change, the behavior of the cell, e.g., the way they spread, changes as well [8]. In human cancer cell lines, invasive cells exhibit biophysical properties that are distinct from their noninvasive counterparts with a reduction in stiffness and an increase in metastatic efficiency [2,3,4,9,10,11], especially in the case of circulating tumor cells (CTCs). Therefore, the biophysical properties of cells can be considered as biomarkers to distinguish cells and thereby used as practical label-free indicators for routine clinical examinations targeting early disease diagnosis.

Numerous methods have been used to examine the biophysical properties of single cells: biomechanical assays to probe cell components [3,12,13] or single cell deformation [10,14,15,16], cytoadherence assays [8], and microfluidics-based assays for mechanical properties [5,17] or for electrical properties [18]. As the majority of these methods can be applied solely for the study of adherent cells, suitable options to analyze non-adherent cells, such as CTCs, are limited. Moreover, there is a trade-off between throughput and information content [7]. Certain techniques such as atomic force microscopy (AFM) allow very sensitive measurements but suffer from low-throughput, while microfluidics-based methods like deformability cytometry provide rapid measurements but with limited information content. However, using biophysical properties for practical routine tests to distinguish cells in a sample solution requires both high-throughput and multi-parameter analysis.

Three main features necessary for the targeted practical system are (i) the high-throughput handling of non-adherent cells without compromising (ii) the detection sensitivity for (iii) a multi-parameter analysis. Microfluidics technology is well-suited to handle non-adherent cells and such capabilities have already been demonstrated with high-throughput techniques [5,17]. For a practical yet still highly sensitive multi-parameter detection method, on the other hand, microelectromechanical systems (MEMS) technology provides low-noise electrical detection while providing mechanical and electrical stimulation of cells. Integrating MEMS with microfluidics enables the handling and/or analysis of biological samples such as microtubules [19], DNA [20], and cells [21], even for clinical purposes [22]. MEMS devices with built-in microfluidics simplify biophysical characterization and improve throughput, even though existing examples use external actuation and optical detection [23,24]. To achieve the optimal use of MEMS, sensitive harmonic analysis can be combined with practical electrostatic actuation. However, to reach the necessary sensitivity, the MEMS elements have to be operated in air while the cell characterization is performed in liquid.

This study develops a MEMS device with an embedded microfluidic channel to perform multi-parameter biophysical (mechanical and electrical) characterization. Electrostatic actuation allows both single cell stimulation and harmonic analysis for sensitive real-time measurements due to a specific device design, separating the in-liquid biological sample handling from in-air MEMS actions (actuation and detection). Here, we introduce the device features, perform real-time measurements (mechanical and electrical), and demonstrate a proof of concept for single cell handling. This device will allow high-throughput characterization of individual cells in complex biological samples. This could open potential tool alternatives for routine clinical tests for early disease diagnosis with improved cost and time efficiency.

## 2. Concept and Design

### 2.1. Principle

**Figure 1:**
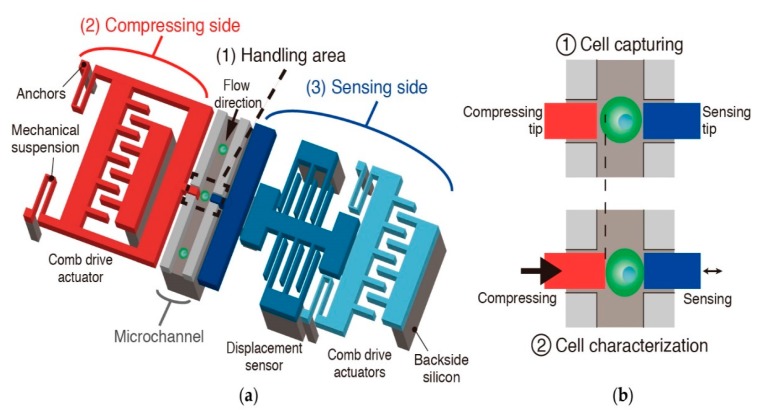
(**a**) Schematic image of the device with embedded channel, (**b**) A close-up view of the tips at the handling area.

The proposed single cell characterization device consists of three main components (Figure 1): (1) a microfluidic channel integrated with two opposing tips to handle cells, (2) a series of comb drive actuators to perform compression, and (3) a differential capacitive sensor for displacement measurements. This sensor is connected to a second electrostatic comb drive actuator for harmonic measurements. A flow is created in the microfluidic channel (shown in gray) with a vacuum pump connected to the outlet after introducing a cell solution via the inlet. Adjusting the vacuum pump pressure enables control of the flow rate, which allows for single cell positioning at the characterization area between tips. One tip, connected to an electrostatic actuator for displacement (shown in red), is used for compressing cells, whereas the other (shown in blue) is used for sensing the mechanical properties of cells under various compressive strain values with a displacement sensor based on a differential capacitor (shown in light blue). After being introduced *via* the inlet, a cell is positioned at the characterization area for opposing tips to perform single cell mechanical/electrical characterization. By controlling the flow rate, the captured cell can then be washed away to receive the next cell for characterization. 

### 2.2. Design Description

**Figure 2:**
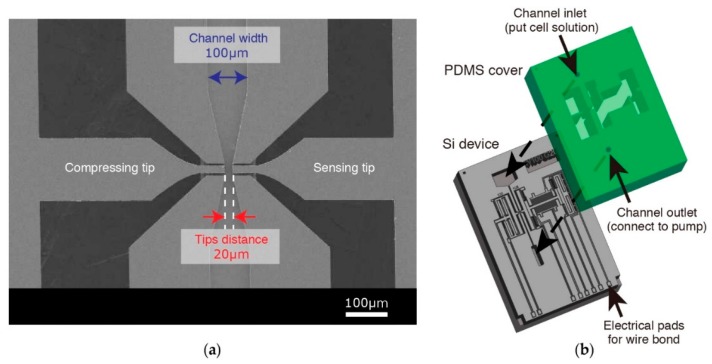
(**a**) Scanning electron microscope (SEM) image of the handling area where compressing and sensing tips access the microfluidic channel, (**b**) Schematic view of polydimethylsiloxane (PDMS) cover alignment.

The device is composed of a MEMS layer and a polydimethylsiloxane (PDMS) layer. The MEMS layer is fabricated on a silicon-on-insulator (SOI) wafer. The top silicon of the wafer forms the functional part of the proposed device (Figure 2a). In addition to being the handling layer of the device, the bottom silicon has two other functions: (i) providing mechanical connection between electrically isolated elements of the sensing part and (ii) completing the microfluidic channel together with the PDMS layer (Figure 2b). The functionality of the device is achieved through the combination of three distinct areas. 

#### 2.2.1. Handling Area and Microfluidic Channel

The handling area consists of a channel formed by the frontside silicon (30 µm) of the SOI wafer as sidewalls, the backside silicon of the SOI wafer as the bottom surface, and the PDMS slab as the top surface. The width of the channel is 100 µm with a narrower portion of 20 µm between the actuation and sensing tips (Figure 2a). The handling channel is accessed by four openings: an inlet and an outlet in the PDMS slab to handle the liquid in the channel (Figure 2b) and two side openings at the sidewalls for the compressing and sensing tips. As both tips need to be moveable, the PDMS surface above the characterization area is 10 µm above the top of the sidewalls, preventing any contact with the actuating elements (Figure S1). The tips are free to move after the oxide layer between the top and bottom silicon of the SOI wafer is removed (see fabrication). The width of the elevated PDMS area is designed as 500 µm to allow for simple alignment of the two layers.

The inlet and outlet openings in the PDMS are formed using biopsy punchers (1 mm for the inlet and 0.5 mm for the outlet) and thus, do not require any specific designs. Injection to the inlet is performed using a micropipette, while the outlet is connected to the pump with a tube. The side openings for the actuating and sensing tips, on the other hand, are fabricated on the SOI wafer (see fabrication) and require careful design consideration. Excessively wide openings may compromise detection sensitivity, while a very narrow opening will cause fabrication difficulties. The objective is to prevent liquid within the channel from leaking out of the backside silicon layer border forming the channel. Ultimately a gap of 5 µm between the sidewalls and the tips was chosen. This value is similar to some previous studies using actuation at an air-liquid interface [25]. Another trade-off for a design parameter is related to the width of the tips themselves. The width was chosen as 20 µm to be comparable with the targeted cell dimensions. Larger tip width could improve cell-capturing efficiency, however, etching the SiO_2_ layer under the tips would be more difficult. 

#### 2.2.2. Compressing Side

**Figure 3:**
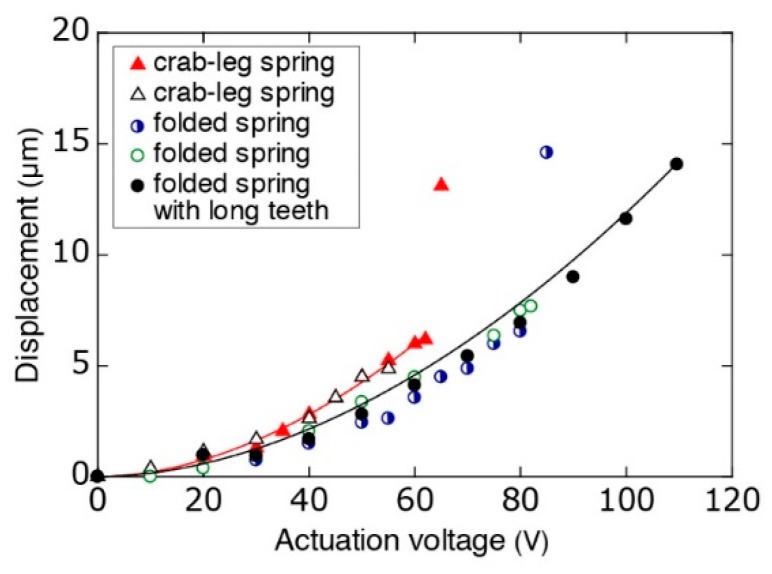
Actuation characteristics of various spring designs.

The compressing side consists of electrostatic comb-drive actuators, which provide the necessary motion of the compressing tip. Depending on the dimension of the targeted cells, the displacement requirement for the actuator varies. Therefore, we use various designs with different spring geometry, spring constant, and actuator teeth dimensions. Two different spring designs are used: crab-leg springs [26] and folded springs [27]. The folded spring design with a spring constant of 40 N·m^−1^ provides highly stable characteristics in terms of y-axis displacement. Conversely, the crab-leg spring design (30 N·m^−1^) is softer than the folded spring design, which allows longer displacement at lower actuation voltages (Figure 3). A wider displacement range is more suitable for use with cells with highly variable diameters. 

**Figure 4:**
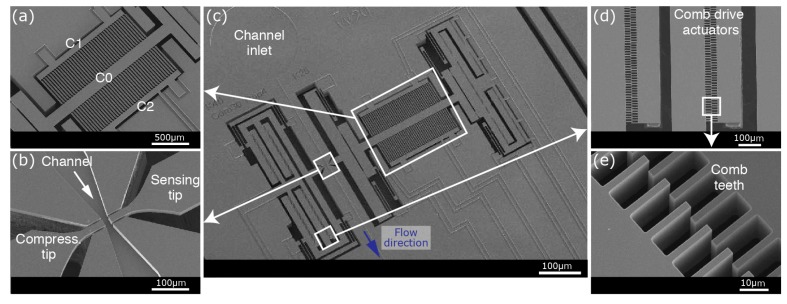
SEM images of the fabricated device: (**a**) Displacement sensor based on differential capacitors, (**b**) opposing tips accessing the handling area, (**c**) an overview of the device, (**d**) electrostatic comb drive actuators with (**e**) a close-up view.

Four groups of comb drives (with 250 teeth per group) provide actuation. Each tooth is designed to have a width of 4 µm. A pitch of 8 µm between each tooth results in a gap of 2 µm between two complimentary opposing teeth (Figure 4). The crab-leg spring design has 20 µm long teeth with 6 µm of overlap between opposing teeth. To obtain a longer stroke with the more-stable folded spring geometry, we use two different designs: 20 µm long teeth (with 4 µm overlap) for ordinary use and 30 µm long teeth (with 4 µm overlap) for applications requiring longer displacement (Table 1, Figure 3).

A typical displacement in a crab-leg spring actuator is 6 µm for an actuation voltage of 60 V. Above 60 V, the comb drive teeth stick to each other. Having a higher spring constant, the folded spring design requires ~75 V to achieve 6 µm of displacement. Devices with a tooth length of 20 µm provide ~8 µm of displacement before suffering from stiction at ~85 V, while the longer teeth length (30 µm) provides displacement beyond 14 µm (at 110 V). 

#### 2.2.3. Sensing Side

The sensing side consists of three elements [26,28]: (i) comb-drive actuators providing the necessary vibrating motion of the sensing tip, (ii) differential parallel plate capacitors for displacement sensing, and (iii) a sensing tip to access the handling area (Figure 1a and Figure 4). These elements are mechanically connected while kept isolated electrically to provide optimal simultaneous mechanical and electrical sensing. The comb-drive actuators are driven by a lock-in-amplifier while the phase is locked based on the output of the displacement sensor for resonance mode.

The actuating elements of the sensing area are suspended with 6 crab-leg springs. Two different sensing area designs were fabricated to provide different stiffness: 5 N·m^−1^ and 25 N·m^−1^. The stiffer design provides enough sensitivity for measurements with harmonic oscillations while static measurements would benefit from the higher sensitivity of the softer design. 

Similar to the compressing actuator, four groups of comb drives (with 280 teeth per group) provide actuation for harmonic detection. Each tooth is designed to have the same dimensions as the crab-leg spring design of the compressing side: a tooth width of 4 µm, a tooth length of 20 µm, a gap of 2 µm and an overlap of 6 µm between two consequent opposing teeth. Although the sensing tip is actuated with amplitude of only 0.2 µm during normal use (3 V_p-p_ actuation voltage), standard 5 µm displacement (at ~50 V) can be provided if required. 

The displacement sensor, comprised of two stationary electrodes with a common movable electrode forming two identical capacitors, works in differential mode [26,29]. Eighty teeth (10 µm × 575 µm; width × length) form parallel-plate capacitors with an initial gap of 40 µm. The movable electrode is designed to have a gap of 5 µm between both stationary electrodes with an overlap of 525 µm (Figure 4). Working in differential mode, the gap between one of the electrode pair increases, while the gap between the other electrode pair decreases during measurements.

### 2.3. MEMS Fabrication Process

**Figure 5:**
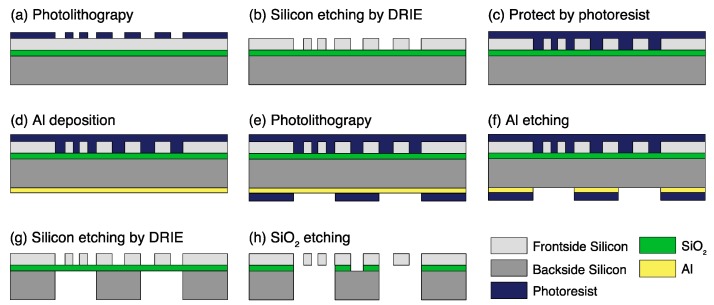
Fabrication process of the device. (**a**) Frontside photolithography, (**b**) Frontside silicon etching, (**c**) Protecting frontside structures, (**d**) Backside Al deposition, (**e**) Backside photolithography, (**f**) Al etching, (**g**) Backside silicon etching, (**h**) SiO_2_ removal.

The device fabrication starts with a standard photolithography process (Figure 5) on the frontside of an SOI wafer (30/2/350 µm-thick). Photoresist is spun on the wafer as a mask for deep reactive ion etching (DRIE), and 30-µm-deep structures (tips, comb drive actuators, capacitive sensors) are defined. After protecting the etched structures on the front surface with a photoresist layer, a 100-nm-thick aluminum (Al) mask is patterned on the backside surface as an etch mask for another DRIE process stopping at the buried oxide layer (BOX) and forming 350-µm-deep structures. The final process is the removal of the SiO_2_ BOX layer with hydrofluoric acid (HF) to release the structures. As a result, the opposing tips are suspended to move freely for single cell characterization (Figure 5). 

The PDMS top cover is fabricated using a standard soft-lithography process. SU8-2015 (with a height of 10 µm, MicroChem, Westborough, MA) is patterned on a silicon wafer and used as a mold for PDMS. Then, the channel inlet and outlet are opened using biopsy punchers (1 mm for the inlet and 0.5 mm for the outlet). Finally, the PDMS cover is aligned and assembled on the MEMS device to form the channel (Figure 2b).

## 3. Setup and Operation

### 3.1. Experimental Setup

**Figure 6:**
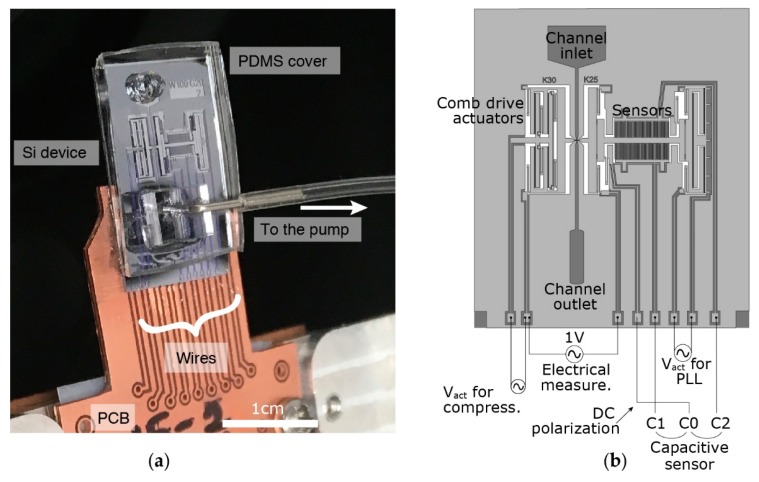
(**a**) An assembled device on printed circuit board (PCB) mounted on the setup, (**b**) Schematic view illustrating electrical connections of a device.

Experiments are performed on an upright microscope stage (VH-S30B, Keyence Corporation, Osaka, Japan). A long-working distance objective (VH-Z50L, Keyence Corporation) and a camera (Infinity 3, Lumenera Corporation, Ottawa, ON, Canada) are used to monitor the experiments. The fabricated devices are connected to peripheral electronics and fluidic equipment, which are controlled and driven by a LabVIEW (version 16, National Instruments Corporation, Austin, TX, USA) program. Prior to positioning on the microscope stage, the assembled device is mounted on a printed circuit board (PCB) and connected with aluminum wires (Figure 6a). 

Actuating the compressing tip requires applying a potential difference between the combs of the electrostatic actuator. This potential difference is provided with a function generator (33500B, Keysight Technologies Inc., Santa Rosa, CA, USA) and a high voltage amplifier (WMA-100, Falco Systems BV, Katwijk aan Zee, The Netherlands) to maintain higher voltages. As one of the actuation electrodes is also used for electrical sensing, this electrode is grounded virtually by the transimpedance amplifier needed for electrical measurements and the actuation signal is applied on the other electrode (Figure 6b).

The sensing side has a more complex structure to provide mechanical oscillations for harmonic analysis while simultaneously performing electrical measurements. Phase-lock loops (PLL) are essential to perform harmonic oscillations for mechanical measurements. A lock-in-amplifier (Model 7230, AMETEK, Inc., Berwyn, PA, USA) drives the comb-drive actuations at a constant phase according to the differential capacitive sensor readings. Two stationary electrodes (C_1_ and C_2_), forming two identical capacitors with the movable electrode (C_0_), are connected to the inputs of the lock-in-amplifier after passing through low-noise current-to-voltage (A/V) preamplifiers (Signal Recovery, model 5182). C_0_, on the other hand, is connected to a power source (provided by the lock-in-amplifier) to be polarized with a constant voltage. The lock-in-amplifier uses sensor measurements to drive the actuators at the resonance frequency. The tip of the sensing area is connected to another lock-in amplifier (HF2LI, Zurich Instruments Ltd., Zurich, Switzerland) for electrical measurements. An electrical signal is applied on the sensing tip, while the compressing tip is connected to a transimpedance preamplifier (Zurich Instruments HF2TA) that feeds the input of the lock-in amplifier. 

The channel outlet on the microfluidic PDMS cap is connected to a flow sensor with tubing before reaching the vacuum pump (AF1, Elveflow, Paris, France, Figure 6a). This equipment is monitored and controlled by the LabVIEW program.

### 3.2. Liquid Handling

As cell characterization can only be performed in a dedicated area, targeted cells inserted via the channel inlet have to be transported and positioned between the tips. Applying a negative pressure at the outlet of the channel creates a flow and adjusting the pressure level changes the flow speed to control the motion of cells in the channel. 

**Figure 7:**
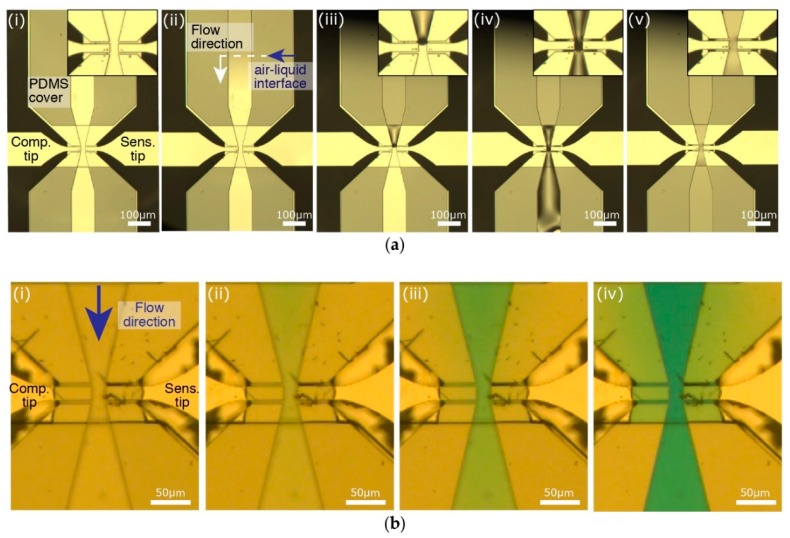
(**a**) After assembling the PDMS cover on the MEMS device (i); the formed channel is filled with liquid. Adjusting the pressure with the pump, the liquid enters the channel (ii); reaches the opposing tips (iii); goes through the handling area (iv); and finally, completely fills the channels; (**b**) The liquid exchange capability of the device is tested with water and a blue dye solution. (i) While the channel is filled with water; (ii,iii) a blue dye solution is injected at the inlet and within seconds (iv) the liquid in the channel is completely replaced.

After assembling the PDMS cover and the MEMS device, we connect the outlet of the channel to the pump with tubes (Figure 7ai). At first, we fill the channel and the connection tube with water (Figure 7aii–iv). Due to the small dimensions (5 µm gap between tips and the sidewalls), high surface tension at the handling area maintains the stability of the air-liquid interface. As a result, the liquid inside the channel does not leak out (Figure 7av). Moreover, by injecting another solution in the inlet, we can exchange the solution in the channel within seconds [20,30]. This property is important for a variety of purposes, for example washing, surface treatment, and drug testing. This is demonstrated by replacing water inside the channel with a blue-dye solution (Figure 7b).

### 3.3. Mechanical Detection Method

By avoiding the usage of the MEMS elements in liquid, we achieve a higher system quality factor when performing sensitive harmonic analyses [26,29]. In short, the sensing part of the device oscillates harmonically with a series of comb-drive actuators (0.2 µm of actuation at 3 V_rms_). These actuators are driven at the resonance frequency of the system by a lock-in amplifier (Signal Recovery, AMETEK 7270 DSP). Due to the mechanical connection (between the sensor, the actuator and the sensing tip), actuators oscillate the central electrode (C_0_) of the differential capacitive sensor. Detecting the capacitance between the movable common electrode (C_0_) and the other two fixed electrodes of the differential capacitive sensor (C_1_ and C_2_), a LabVIEW-controlled PLL keeps the sensing arm at resonance throughput the experiment. Capturing a biological sample between the tips changes the spring constant of the system, which can be detected as a change in the resonance frequency by the PLL driven by the lock-in amplifier.

To improve the detection performance of the displacement sensor, C_0_ is polarized with a constant voltage V_0_ (3 V) and the fixed electrodes are kept grounded [28]. During harmonic oscillations, the change in the gap between the mobile and fixed parallel plate electrodes creates dynamic currents that are collected from C_1_ and C_2_. Amplified with low-noise current-to-voltage preamplifiers (with a gain of 10^8^), the sensor outputs are fed into the lock-in-amplifier for real-time measurements.

### 3.4. Electrical Detection Method

Electrical insulation of the various elements of the sensing area is crucial for simultaneous mechanical and electrical detection. By connecting through the backside silicon, the SiO_2_ layer keeps the sensing elements mechanically attached while providing electrical access to each individual element. This allows us to apply a sinusoidal signal at the actuator (3 V_rms_ at the resonance frequency of the system) and to read the sensor outputs in parallel with the electrical measurements performed at the tip of the sensing arm.

The electrical detection is performed between the sensing and the compressing tips. A 1 V_p-p_ signal is applied on the sensing tip, while the compressing tip is connected to the virtual ground of a transimpedance amplifier. The dynamic current passing through the amplifier is converted to voltage and measured in real-time. Moreover, by sweeping the frequency at the sensing tip, the frequency response of the system can be analyzed. 

## 4. Device Performance and Results

Hereafter, we show some results to investigate the performance of the proposed device. Starting with the frequency response of the device, we performed mechanical and electrical measurements using different sample solutions. Finally, we demonstrated the biomaterial-handling capabilities of the proposed device to confirm the capability of single cell analysis.

### 4.1. Frequency Response and Real-Time Analysis

**Figure 8:**
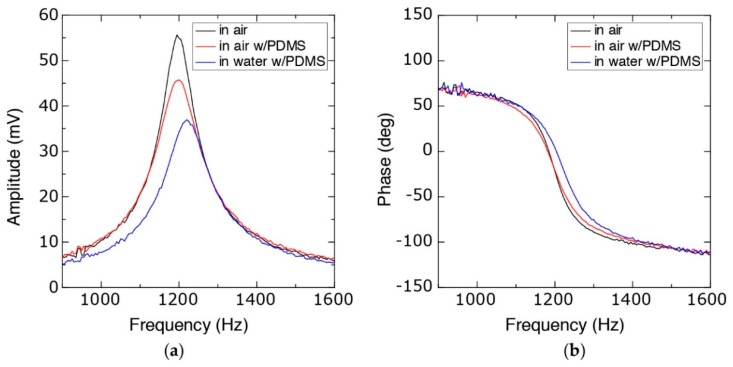
Frequency response of the device in different conditions showing (**a**) the amplitude and (**b**) the phase shift of the sensor readouts.

To characterize the behavior of the device during harmonic analysis, the frequency response of the system was monitored with the lock-in-amplifier (Figure 8). Compared to the initial device characteristics (resonance frequency of 1195 Hz with a quality factor (Q-factor) of 8.9), the assembled device (with PDMS cover) showed a slight increase in the resonance frequency (1200 Hz) and a decrease in the Q-factor (6.3) due to the increased damping as a result of the PDMS slab. Filling the channel with liquid also changes the frequency response. Although the effect of the liquid in terms of mass is negligible compared to the total mass of the mobile part of the sensing arm, the surface tension due to the air-liquid interface in the handling region did affect the spring constant of the system. As a result, the resonance frequency was increased to 1220 Hz with a similar Q-factor (6.8). 

Observing the changes in the mechanical properties of biological samples requires real-time monitoring of the system response. To achieve this, we performed repeated PLLs controlled by a LabVIEW program. Before comparing different samples and solutions, we tested the stability of the detection method. Although the mechanical characterization of cells requires less than 30 s, we monitored the system for over five min (Figure S2). Confirmation of the system stability allowed us to perform mechanical measurements in various sample media. 

### 4.2. Mechanical Measurements

Mechanical detection performance of the system was tested by measuring the change in the resonance frequency in solutions with different viscosities and surface properties. We compared the mechanical response of the device in glucose solutions of various concentrations ranging from 0.1% to 10% (*w*/*v*). Using the vacuum pump, these solutions were consecutively injected after filling the channel with water. The PLL measurements allowed real-time observation of the changes in the solution between the tips. 

**Figure 9:**
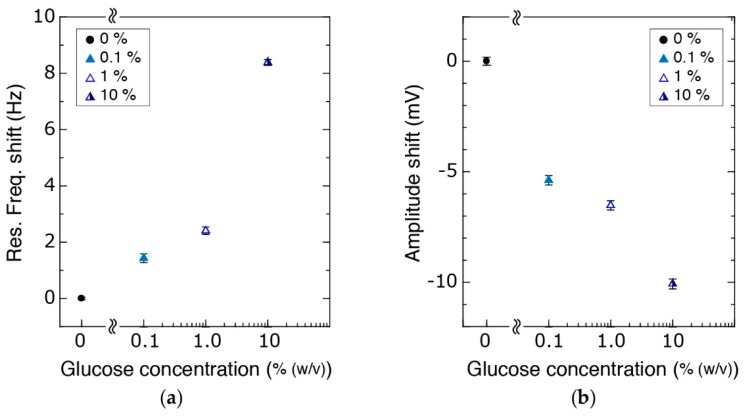
(**a**) Resonance frequency of the system increased with increasing glucose concentration while (**b**) the amplitude decreased.

Increasing glucose concentration resulted in an increase of the resonance frequency (Figure 9). Taking the water measurements as the base value, we obtained a resonance frequency shift of 1.4 Hz, 2.4 Hz, and 8.3 Hz for glucose concentrations of 0.1%, 1% and 10%, respectively (Figure 9a) with stable characteristics (Figure S3). Similarly, a decrease in amplitude is observed with increasing glucose concentration (Figure 9b, Figure S4). Compared to the initial water measurements, a decrease of 5 mV, 7 mV, and 10 mV in the amplitude was observed due to higher damping. These measurements show that the proposed device is capable of observing changes in the mechanical characteristics at the handling area. 

### 4.3. Electrical Measurements

**Figure 10:**
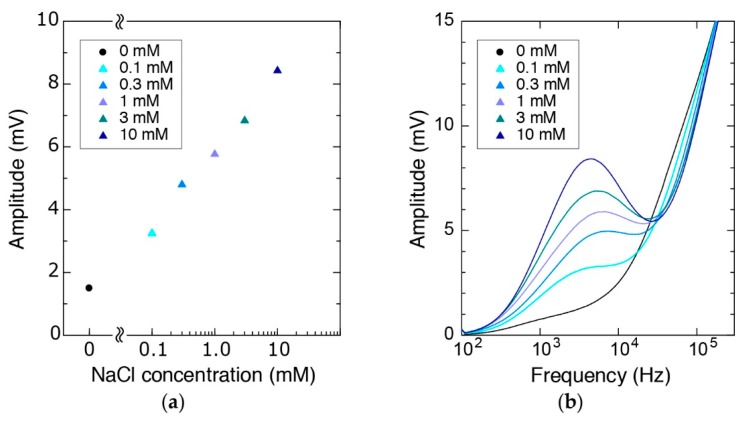
The amplitude of the signal (**a**) at 4 kHz and (**b**) during sweeping the frequency in different conditions.

An important property of the proposed device is the ability to perform electrical measurements together with mechanical characterization. To demonstrate this capability, we injected solutions with different ionic strengths in the channel and examined the electrical properties. Similar to the glucose measurements, we filled the channel first with water and then injected different molar concentrations of NaCl (0.1 mM–10 mM) consecutively. Using a lock-in-amplifier, we applied 1 V_p-p_ to the sensing tip and amplified the signal obtained from the compressing tip with a transimpedance amplifier (with a gain of 10^5^). Measurements resulted in increasing voltage waveform amplitudes for increasing ion concentration when the potential difference was applied at 4 kHz (Figure 10a) indicating an increase in the current passing through the tips. In addition to these real-time measurements (Figure S5), we could also obtain the frequency response of the system by sweeping the frequency from 100 Hz to 100 kHz (Figure 10b, Figure S5). 

### 4.4. Biological Sample Handling 

Besides the mechanical and electrical measurements, a key feature of the proposed device is the ability to handle biological samples, e.g., single cells. We examined the single cell handling capability of the device by injecting solutions of fixed cancer cells. The human SUM159PT breast cancer cell line was purchased from Asterand (Detroit, MI, USA). The cells were cultured in F12 medium (Invitrogen Corporation, Carlsbad, CA, USA) supplemented with 5% fetal bovine serum (Lonza Group, Basel, Switzerland), streptomycin (100 μg/mL), penicillin (100 units/mL), insulin (5 μg/mL) and hydrocortisone (1 μg/mL) (Invitrogen). Prior to the experiments, subconfluent cell culture were trypsinized, resuspended in single cell solution, and then fixed with 4% Paraformaldehyde (10 min, RT), and rinsed with phosphate buffered saline (PBS).

**Figure 11:**
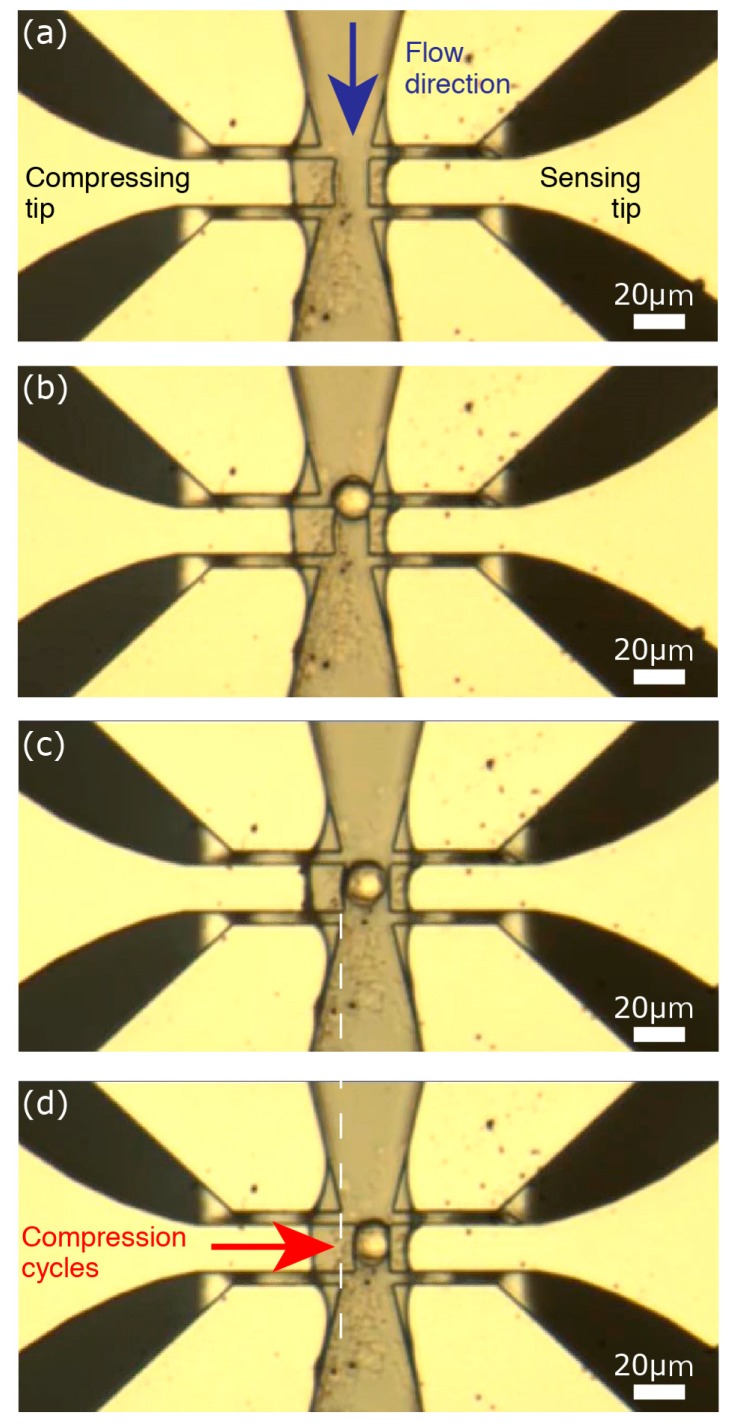
Sequential photos demonstrate single cell capturing. (**a**) Applying a potential difference between the compressing actuator electrodes narrows the gap between the tips; (**b**) The solution is kept flowing until a cell arrives at the handling area; (**c**) Then, the flow rate and the potential difference between the compressing actuator electrodes are decreased until the cell is positioned between the tips; (**d**) Finally, the flow is completely stopped and cell compression is performed.

We controlled the vacuum pump and the compressing actuator to capture single cells. After injecting the cell solution in the channel, we created a flow with pump and applied 110 V at the compressing actuator electrodes to narrow the gap between the tips. This decreases the width of the channel from 20 µm to ~6 µm (Figure 11a). Thus, cells with a mean diameter of ~16 µm could not pass through. When a cell was stopped at the handling area (Figure 11b), we immediately stopped the flow and decreased the applied voltage to widen the gap between the tips and position the cell between the tips (Figure 11c). With the cell thusly captured, we could then apply the compression signal at the compressing actuator (Figure 11d) to examine the characteristics of the captured cell. After measurements, the compressing tip was moved back to the initial (open) position and the flow was restarted to remove the captured cell and prepare for another cell to characterize. 

An important point to note was the stability of the air-liquid interface at the sensing tip. Although the compressing arm moved towards the sensing tip while compressing the cell, the air-liquid interface at the sensing tip was not affected. This stability is a crucial point for reliable and sensitive detection with the proposed device.

## 5. Conclusions

Separating handling and sensing elements of a microfabricated device is the key to handle and analyze biological samples without compromising precise MEMS performance. We demonstrated a MEMS device with a built-in microfluidic channel to perform single cell biophysical characterization. The built-in channel, requiring no assembly actions between the MEMS and microfluidics elements, not only provides higher-throughput for analysis but also improves sensitivity by allowing integration of microfluidic and MEMS elements at a much finer assembly resolution. Moreover, simultaneous electrical and mechanical measurements allow various parameters to be targeted, such as size, stiffness, viscous losses, membrane fluidity, membrane capacitance, and cytoplasm resistivity. The device is sensitive enough to distinguish differences between liquids according to their mechanical and electrical properties. As demonstrated with cell compression, the device is capable of capturing a single cell and stimulating it mechanically. Sensitive real-time measurements and highly controllable mechanical stimulation allow single cell characterization at different compression levels. Together with the electrical measurements, the device allows for practical multi-parameter analyses of single non-adherent cells to perform routine clinical tests for early disease diagnosis with improved cost and time efficiency.

## Figures and Tables

**Table 1:** Main parameters of device designs.

Device	Compressing Side			Sensing Side	
	Spring Shape	Spring Constant (N/m)	Comb Tooth Length/Overlap (µm)	Spring Shape	Spring Constant (N/m)
Design 1	Crab-leg	30	20/6	Crab-leg	25
Design 2	Folded	40	20/4	Crab-leg	5
Design 3	Folded	40	30/4	Crab-leg	25

## Supplementary Materials

The following are available online at http://www.mdpi.com/2072-666X/9/6/275/s1, Figure S1: Schematic view of the assembly, Figure S2: Real-time stability measurements, Figure S3: Real-time frequency monitoring in glucose solutions with different concentrations, Figure S4: Real-time amplitude monitoring in glucose solutions with different concentrations, Figure S5: Real-time electrical measurements.
