# Supplementary material for: Developing a MEMS Device with Built-in Microfluidics for Biophysical Single Cell Characterization
Source: Micromachines (Basel). 2018 Jun 1;9(6):275. doi: 10.3390/mi9060275 (PMC6187549; doi:10.3390/mi9060275)
Supplement: Supplementary file 1 [file micromachines-09-00275-s001.pdf]

Supplementary Materials: Developing a MEMS Device with Built-in Microfluidics for Biophysical Single Cell Characterization

Yuki Takayama, Grégoire Perret, Momoko Kumemura, Manabu Ataka, Samuel Meignan, Stanislav L. Karsten, Hiroyuki Fujita, Dominique Collard*, Chann Lagadec and Mehmet Cagatay Tarhan *

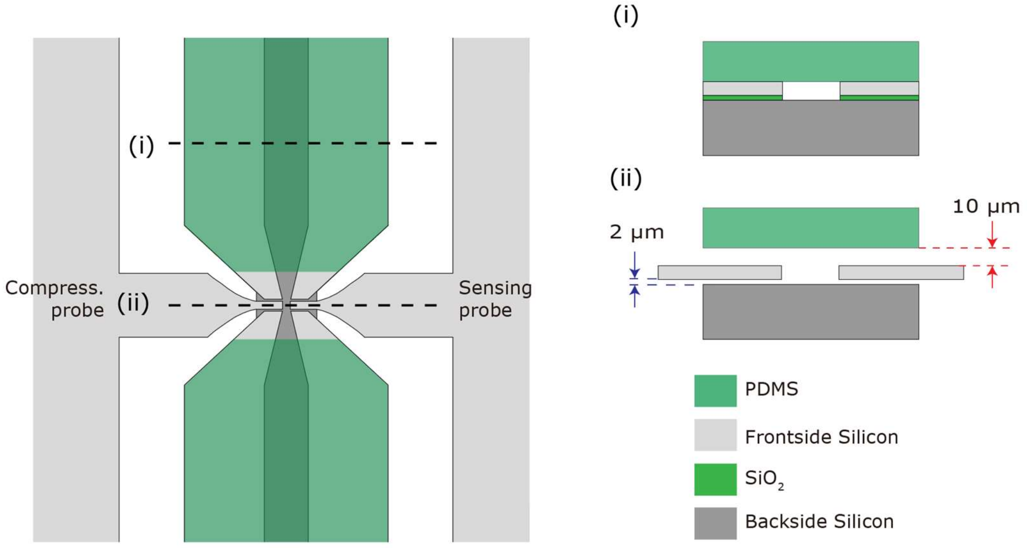

(**a**) (**b**)

Figure S1: Schematic view of the microelectromechanical systems (MEMS) and polydimethylsiloxane (PDMS) cover assembly around the handling area. (**a**) The top view shows the attachment area between the PDMS cover and the MEMS device surface. (**b**) The side view shows two different cross-section: (i) The formed channel between the MEMS device and the PDMS cover is completely closed apart from (ii) the handling area where removal of SiO_2 layer and the higher PDMS bottom surface allow movable parts of MEMS device to move freely.

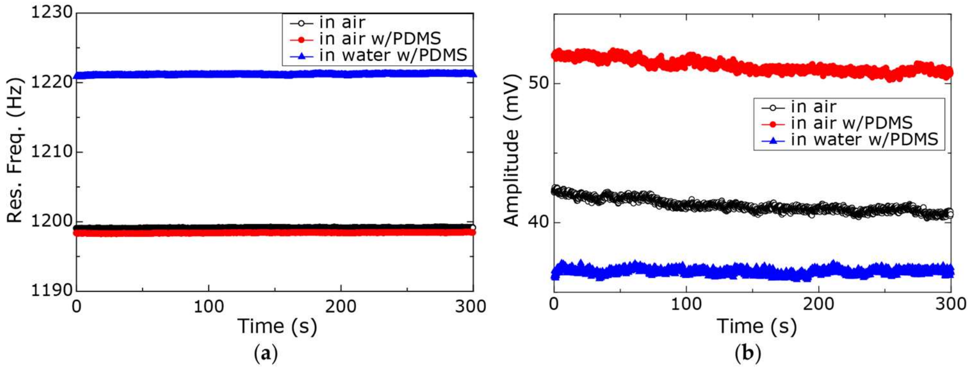

(**a**) (**b**)

Figure S2: Real-time measurements of the device to examine the detection stability in terms of (**a**) resonance frequency, and (**b**) amplitude.

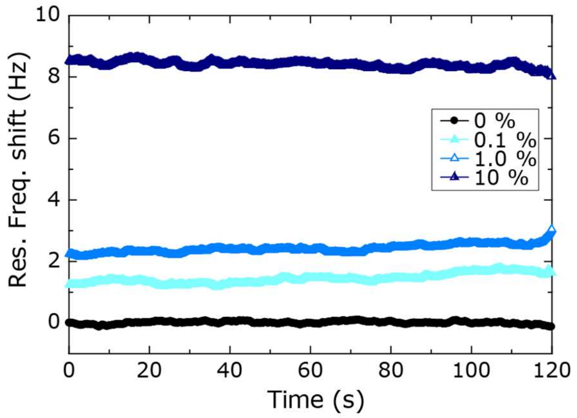

Figure S3: Real-time measurements showing the mechanical characteristics of glucose solutions with different concentrations. Resonance frequency increased with increasing glucose concentration.

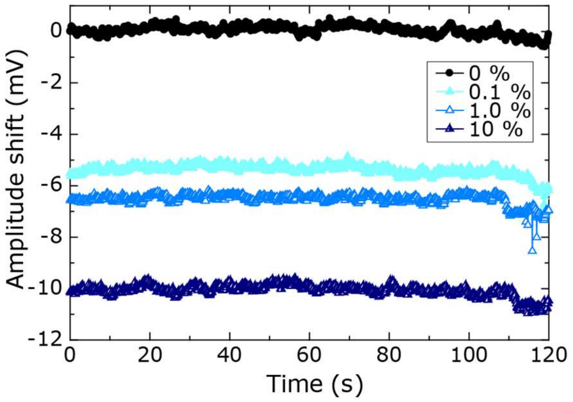

Figure S4: Real-time measurements showing the mechanical characteristics of glucose solutions with different concentrations. Amplitude decreased with increasing glucose concentration.

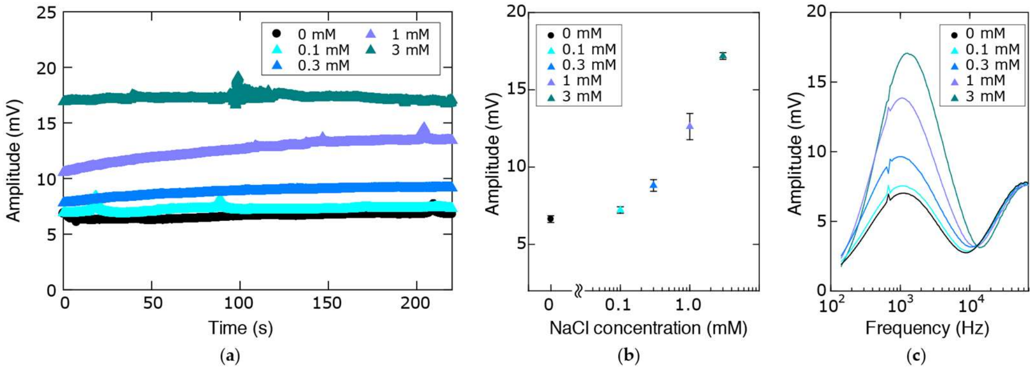

Figure S5: A set of electrical measurements performed in NaCl solutions with different concentrations. (**a**) The real-time measurements were performed at 1 kHz. Higher NaCl concentration resulted in a higher current which is detected as higher amplitude. (**b**) Average value of the real-time measurements show the relation between the NaCl concentration and the amplitude. (**c**) Sweeping the frequency provided the frequency response of the system. Note that the electrical measurements detected a small noise due to the mechanical measurements at around 600 Hz. The target frequency for the electrical measurements and the mechanical resonance frequency of the MEMS device should be designed carefully.
